# A Case of Poisoning by Duboisia

**Published:** 1880-11

**Authors:** E. L. Holmes

**Affiliations:** Chicago


### ARTICLE VI.

### *I. A Case of Poisoning by Duboisia. II. A New Mydriatic—Homatropin.* By E. L. HOLMES, of Chicago.

(Read before the West Chicago Medical Society.)

I. Mr. L. B., sixty-five years of age, in excellent health, a patient at the Illinois Charitable Eye and Ear Infirmary, during convalescence after Graefe's operation for cataract, was provided with a small bottle of sulphate of duboisia, gr. j to ℥ j, in place of sulphate of atropia, which caused considerable conjunctival inflammation.

On the 27th of April, about 9 o'clock in the evening, Mr. B. took by mistake a "teaspoonful" of the solution. It cannot be determined whether the teaspoon was quite full.

The patient at once informed other patients near him that he had taken the wrong solution, but concluded to await the result, before reporting to the nurse. In about ten minutes there was dryness of the throat, and in half an hour a peculiar sensation in the legs, then in the thighs, arms and other parts of the body, as if they were asleep. At the end of three-quarters of an hour or more, the patient could scarcely talk or stand. Strange to say, not till this time did it occur to the patient or those around him to call the nurse.

An active emetic was at once given with the apparent effect of entirely relieving the stomach of its contents. Without delirium the patient rapidly passed into a state of unconsciousness, and remained in this condition till about five o'clock in the morning. He complained for two days of muscular weakness in the legs and arms, and especially noted a peculiar jerking action of the muscles of the arm in extending the hand to grasp a glass or other object.

I saw the patient at midnight. He was lying quietly, breathing naturally, but in a stupor from which he could not be aroused. The face was not specially flushed, although the mouth and tongue were remarkably dry. Dr. B. W. Griffin, who had been with the patient two hours, informed me that the temperature, as determined by the thermometer, had been normal. The pulse varied from 108 to 112. Some time previous to this, before unconsciousness became quite complete, the patient made ineffectual efforts to sit up in bed. The pulse always fell to 80 when the patient was supported in a sitting posture, and increased in frequency again on reclining. No other symptoms were noticed.

About an ounce of brandy in water was administered in minute doses during the night. Morphine was not given as an antidote.

I have observed four cases of serious atropine poisoning, in all of which wild delirium was a very prominent symptom.

It is claimed as a chief advantage in the use of duboisia that it scarcely ever causes irritation of the conjunctiva and skin which not unfrequently attends the use of atropia. I have seen one case, however, in which duboisia produced these troublesome symptoms. This agent and its properties are now quite extensively described in the recent works on materia medica and therapeutics. I need not dwell on the subject here.

It may not be out of place, however, to state that Prof. Ladenburg, of Kiel, claimed, some time since, to have demonstrated that duboisin, hyoscyamin and daturin have the same chemical composition and properties. "From these investigations it appears that atropin and hyoscyamin are the only two mydriatic alkaloids. These two, although not identical, are isomeric in composition and otherwise very closely related."

II. Prof. Ladenburg has quite recently discovered a new mydriatic which he calls "homatropin."

Writers in different journals state that this substance is an active mydriatic and paralyzer of the accommodation, with the advantages that it does not irritate the conjunctiva and integument and is not very poisonous. Its effects subside in a few hours, while those of atropine continue ten or even fourteen days.

I have not had access to papers on the chemical properties of this new agent, but am indebted to Prof. W. S. Haines for this brief account of them. He states that Prof. Ladenburg has made a particular study of atropine and its derivatus. He has obtained several alkaloids from atropine, which he calls tropeines. Homatropine is prepared by the union of tropeine and amygdalic acid.

